# Identification of transcription-factor genes expressed in the Arabidopsis female gametophyte

**DOI:** 10.1186/1471-2229-10-110

**Published:** 2010-06-16

**Authors:** Dongfang Wang, Changqing Zhang, David J Hearn, Il-Ho Kang, Jayson A Punwani, Megan I Skaggs, Gary N Drews, Karen S Schumaker, Ramin Yadegari

**Affiliations:** 1 School of Plant Sciences, University of Arizona, Tucson, Arizona 85721-0036, USA; 2 Department of Biology, University of Utah, Salt Lake City, Utah 84112-0840, USA; 3 Current Address: The Section of Molecular, Cell and Developmental Biology, University of Texas at Austin, Austin, Texas 78712-0159, USA; 4 Current Address: Department of Biological Sciences, Towson University, Towson, Maryland 21252-0001, USA; 5 Current Address: Department of Horticulture, Iowa State University, Ames, Iowa 50011-1100, USA; 6 Current Address: Department of Biology, University of North Carolina at Chapel Hill, Chapel Hill, North Carolina 27599-3280, USA

## Abstract

**Background:**

In flowering plants, the female gametophyte is typically a seven-celled structure with four cell types: the egg cell, the central cell, the synergid cells, and the antipodal cells. These cells perform essential functions required for double fertilization and early seed development. Differentiation of these distinct cell types likely involves coordinated changes in gene expression regulated by transcription factors. Therefore, understanding female gametophyte cell differentiation and function will require dissection of the gene regulatory networks operating in each of the cell types. These efforts have been hampered because few transcription factor genes expressed in the female gametophyte have been identified. To identify such genes, we undertook a large-scale differential expression screen followed by promoter-fusion analysis to detect transcription-factor genes transcribed in the Arabidopsis female gametophyte.

**Results:**

Using quantitative reverse-transcriptase PCR, we analyzed 1,482 Arabidopsis transcription-factor genes and identified 26 genes exhibiting reduced mRNA levels in *determinate infertile 1 *mutant ovaries, which lack female gametophytes, relative to ovaries containing female gametophytes. Spatial patterns of gene transcription within the mature female gametophyte were identified for 17 transcription-factor genes using promoter-fusion analysis. Of these, ten genes were predominantly expressed in a single cell type of the female gametophyte including the egg cell, central cell and the antipodal cells whereas the remaining seven genes were expressed in two or more cell types. After fertilization, 12 genes were transcriptionally active in the developing embryo and/or endosperm.

**Conclusions:**

We have shown that our quantitative reverse-transcriptase PCR differential-expression screen is sufficiently sensitive to detect transcription-factor genes transcribed in the female gametophyte. Most of the genes identified in this study have not been reported previously as being expressed in the female gametophyte. Therefore, they might represent novel regulators and provide entry points for reverse genetic and molecular approaches to uncover the gene regulatory networks underlying female gametophyte development.

## Background

The female gametophyte is an integral component of the plant life cycle and plays an essential role in plant reproduction. In most angiosperms including Arabidopsis, the female gametophyte (also called the embryo sac) typically consists of an egg cell, a central cell, two synergid cells, and three antipodal cells [1,2]. During double fertilization, the pollen tube penetrates one of the synergid cells and releases two sperm cells, which fuse with the egg cell and the central cell to give rise to the embryo and the endosperm, respectively [3]. The embryo forms the next generation while the endosperm functions to support embryo development and/or seedling development after seed germination [4-6]. Normal seed development depends on the proper differentiation and functions of the central cell and the egg cell [1,2,7-20]. Although the synergid cells do not directly contribute to the development of the seed after fertilization, they are required for pollen tube attraction [21-26] and proper discharge of pollen tube contents [27-31]. In contrast to the other cell types of the female gametophyte, no clear function has been found for the antipodal cells thus far.

In Arabidopsis and most other angiosperm species, the development of the female gametophyte follows a monosporic, Polygonum-type developmental pattern, in which a single functioning megaspore undergoes three rounds of mitosis without cytokinesis, eventually producing a seven-celled structure with the egg cell and the two synergid cells at one pole (micropylar pole), three antipodal cells at the opposite pole (chalazal pole), and a large central cell in the center [1,2,32]. As in many other developmental processes [33], cell differentiation in the female gametophyte is likely under the control of gene-regulatory networks that consist of transcription factors and their downstream targets. Therefore, identification of transcription-factor genes expressed in the female gametophyte is an important step towards understanding female gametophyte development.

Much of our knowledge about female gametophyte development has been obtained through the analysis of over 100 female gametophyte mutants [1,2,7,9,15,20,34-44]. However, only four of these mutants have been clearly shown to be affected in transcription-factor genes: *agl80 *and *agl61 *mutants are defective in central cell specification and development [7-9], *myb98 *mutants show defects in synergid cell development and function [22,25,26], and *agl23 *mutants are defective in megagametogenesis [43]. Transcription-factor genes involved in the specific development of the egg cell or the antipodal cells have not been identified.

Expression-based analyses have also been used to identify many genes expressed in the female gametophyte. For example, cDNA libraries from isolated egg cell, central cell, synergid cells, or whole embryo sacs, have led to the identification of hundreds of female gametophyte-expressed genes in maize, wheat, tobacco, and *Torenia fournieri *[23,24,45-53]. However, few transcription-factor genes have been reported in these studies. Differential-expression screens using Arabidopsis mutants including *sporocyteless *(*spl*), *determinate infertile 1 *(*dif1*), and *coatlique *(*coa*) that lack female gametophytes have been utilized more recently to identify genes that are expressed in the female gametophyte [54-57]. These mutants are defective either in the initiation of meiosis (*spl*), progression through meiosis (*dif1*), or the initiation of megagametogenesis (*coa*) and therefore do not produce any female gametophytes in an otherwise normal-looking ovule [54,58-60]. In these screens, mRNA profiles from wild-type and mutant ovules or pistils were compared, and genes with reduced mRNA levels (down regulated) in the mutant as compared to wild type would include those that are expressed in the female gametophyte. These microarray-based screens have identified 225 down-regulated genes in *spl *ovules [57], 71 and 382 down-regulated genes in *dif1 *ovules [55,56], and 421 down-regulated genes in *coa *pistils [54]. Despite the large number of genes identified in these screens, only two transcription-factor genes, *MYB98 *[22,56] and *AT5G50915 *[54], have been confirmed to be expressed in the female gametophyte. It is likely that standard microarray techniques are not sufficiently sensitive for the detection of low-prevalence mRNAs typical of most transcription-factor genes [61,62].

Here we report the identification of a large number of transcription-factor genes expressed in the Arabidopsis female gametophyte. Using quantitative reverse-transcriptase PCR (qRT-PCR), we carried out a differential expression screen of 1,482 transcription-factor genes and identified 26 genes down-regulated in *dif1 *mutant ovaries lacking female gametophytes. Localization of transcriptional activities within the female gametophyte was confirmed for 17 transcription-factor genes using *promoter:GFP *fusions. Of these, ten genes are predominantly expressed in a single cell type of the female gametophyte: the egg cell, the central cell, or the antipodal cells. The remaining seven genes are expressed in two or more cell types. We also show that 12 transcription-factor genes are transcribed in the embryo and/or the endosperm of early developing seeds. The majority of these transcription-factor genes have not been previously implicated in female gametophyte development or function. Therefore, our results provide a valuable starting point for elucidating the gene-regulatory networks governing differentiation during female gametophyte development.

## Results

### Quantitative RT-PCR analysis of transcription-factor mRNAs in ovaries

We carried out a differential expression screen to identify transcription-factor genes expressed in the late stages of Arabidopsis female gametophyte development, during which female gametophyte cell types are established [63]. With some modifications, we employed a strategy we previously used to identify genes expressed during female gametophyte development [9,22,56]. In brief, we used qRT-PCR to identify mRNAs with reduced levels in *dif1 *ovaries, which lack female gametophytes [59,60,64], relative to *male sterile1 *(*ms1*) ovaries, which contain wild-type female gametophytes [65-67]. The qRT-PCR reactions were performed with RNA samples obtained from *dif1 *and *ms1 *ovaries harvested from flowers at developmental stages 12C to 14 [68], which, in wild type, contained female gametophytes at developmental stages FG5 to FG7 [63]. We normalized the raw threshold cycle values (C_T_) for each *ms1 *or *dif1 *reaction against C_T _values obtained for *ACTIN2 *(*ACT2*) mRNA (expressed as C_T, *ms1 *_and C_T, *dif1 *_values). We quantified the relative changes in mRNA levels between *ms1 *and *dif1 *ovaries by determining the differences between the normalized C_T, *ms1 *_and C_T, *dif1 *_values (expressed as ΔΔC_T_).

We carried out an initial qRT-PCR screen (referred to as the primary screen) of 1,482 transcription-factor genes (Additional file 1). To compare levels of transcription-factor mRNAs in *ms1 *and *dif1 *ovaries, the normalized single or the averaged C_T _values for each tested transcription-factor mRNA from the *ms1 *and *dif1 *RNA sources were plotted against each other (Additional file 2). The data indicated that the mRNA levels of most transcription-factor genes were not affected in the ovaries by the absence of the female gametophyte [Pearson correlation (*r*) ~ 0.981]. We considered mRNAs with a ΔΔC_T _value above 1.5 or below -1.5 to be differentially elevated in *ms1 *or *dif1 *ovaries, respectively (outside the dashed lines; Additional file 2). By this criterion, 69 mRNAs were elevated in *ms1 *as compared to *dif1 *(ΔΔC_T _above 1.5; Additional file 2) and 58 mRNAs showed an elevated level in *dif1 *(ΔΔC_T _below -1.5; Additional file 2). The latter group likely represents genes whose activity within the ovaries is increased due to the absence of the female gametophyte in the *dif1 *mutant plants and was not studied further. Because the former group of transcription-factor mRNAs represents genes that are likely expressed in the female gametophyte, we selected them for further analysis.

The 69 transcription-factor mRNAs with ΔΔC_T _values above 1.5 in the primary screen were analyzed under more stringent qRT-PCR conditions. For this, we performed three additional sets of qRT-PCR reactions (referred to as the secondary screen) using independently isolated ovary RNAs (Additional file 3). A high level of reproducibility was observed among the three biological replicates from both the *ms1 *(*r *~ 0.948-0.980) and *dif1 *(*r *~ 0.916-0.941) genotypes. We applied an arbitrary threshold (ΔΔC_T _>1.0, *P*<0.1, paired Student's *t*-test) to define genes that display higher levels of mRNA in *ms1 *versus *dif1 *ovaries. As shown in Additional file 3, of the 69 genes tested, 26 genes were confirmed to be down-regulated in *dif1 *ovaries.

### Analysis of transcription-factor gene promoter activities in the female gametophyte

To confirm that the transcription-factor genes identified from the qRT-PCR screens are transcriptionally active in the female gametophyte and to determine their patterns of expression, we generated transgenic Arabidopsis plants carrying *promoter:GFP *fusion constructs for 15 of the 26 transcription-factor genes with ΔΔC_T _values above the statistical threshold of the secondary qRT-PCR screen (highlighted in Additional files 1 and 3; summarized in Additional file 4). In addition, we also tested 9 genes with ΔΔC_T _values below the threshold to evaluate the stringency of the secondary screen (highlighted in Additional files 1 and 3). As discussed below, we detected *promoter:GFP *activity for 14 genes from the former group and three genes from the latter group indicating that our secondary qRT-PCR screen represented a relatively robust assay for identifying female gametophyte-expressed genes.

**Figure 1:**
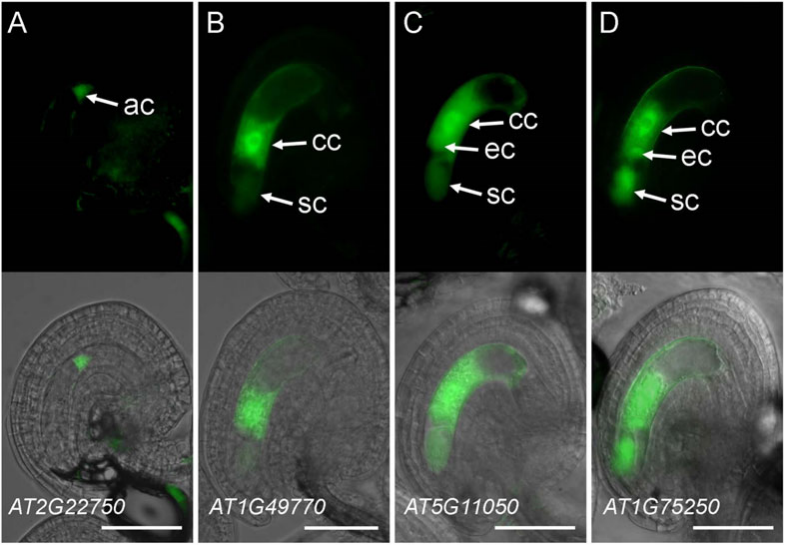
**Expression patterns of transcription-factor *promoter:cGFP *fusions in the female gametophyte**. (A) *pAT2G22750:cGFP *expression in the antipodal cells. (B) *pAT1G49770:cGFP *(*ZOU/RGE1*) expression in the central cell and the synergid cells. (C) *pAT5G11050:cGFP *(*MYB64*) expression in the central cell, the egg cell, and the synergid cells. (D) *pAT1G75250:cGFP *(*RL6*) expression in the central cell, the egg cell, and the synergid cells. Each panel contains an epifluorescence image (top) and an overlay (bottom) of the epifluorescence and a bright-field image of the same ovule. Images in (A) were taken at the developmental stage FG5, the rest of the images were taken at stages FG6 to FG7 [63]. ac, antipodal cells; cc, central cell; ec, egg cell; sc, synergid cell. Scale bars: 50 μm.

**Figure 2:**
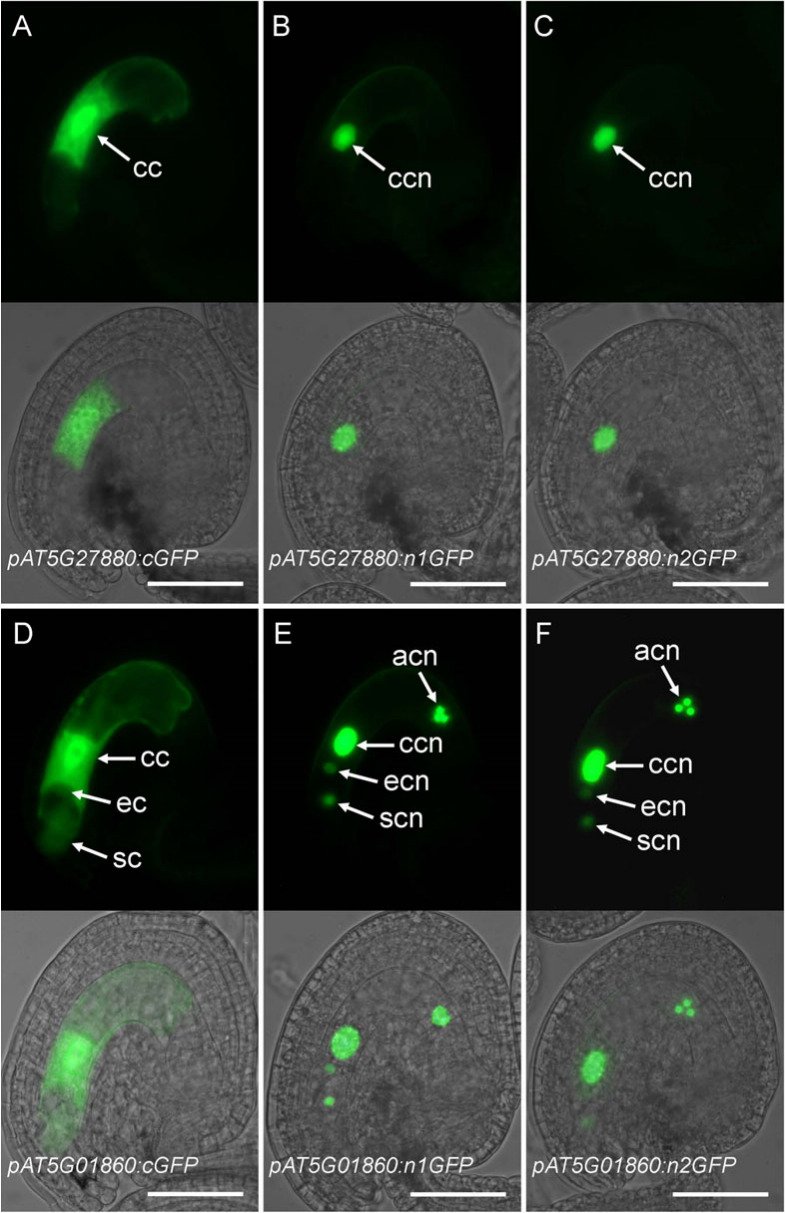
**Comparison of cGFP, n1GFP, and n2GFP gene-reporter activities in the mature female gametophyte**. Expression of *AT5G27880 *(A-C) and *AT5G01860 *(D-F) promoter constructs fused to the *cGFP *(A, D), *n1GFP *(B, E), and *n2GFP *(C, F) reporters. Each panel contains an epifluorescence image (top) and an overlay (bottom) of the epifluorescence and a bright-field image of the same ovule. acn, antipodal cell nuclei; cc, central cell; ccn, central cell nucleus; ec, egg cell; ecn, egg cell nucleus; sc, synergid cell; scn, synergid cell nucleus. Scale bars: 50 μm.

Initially, we generated transgenic plants carrying promoter constructs for six transcription-factor genes using untargeted, cytosolic GFP (cGFP) as a reporter (Table 1, Additional file 5). The *promoter:cGFP *expression patterns were primarily analyzed in the mature female gametophyte. As shown in Figures 1 and 2, all six genes showed patterns of promoter activity in one or more cells of the developing or mature female gametophyte. The activity of the *AT2G22750/bHLH18 *promoter was not detected in mature female gametophytes, but weak GFP activity was detected in the antipodal cells at an earlier stage of development (FG5; Figure 1A). In the mature female gametophyte, GFP activity driven by the *AT5G27880 *promoter was detected exclusively in the central cell (Figure 2A). The other four promoter constructs showed more complex patterns of activity with *GFP *expression detectable in two (*AT1G49770/ZOU/RGE1/bHLH95*; Figure 1B) and three (*AT5G11050/MYB64*, *AT1G75250/RL6*, and *AT5G01860*; Figures 1C, D and 2D) cell types of the female gametophyte. These promoter-fusion expression patterns support the results from our qRT-PCR analysis that these genes are transcribed in the female gametophyte.

**Table 1:** Summary of qRT-PCR and promoter-fusion analyses to identify transcription-factor genes expressed in the female gametophyte.

			Primary qRT screen	Secondary qRT screen		*promoter:GFP *expression	
AGI^1^	Gene family^2^	Gene name^3^	Average ΔΔC_T_	Average ΔΔC_T_	*P *(*t*-test)	Ovule	Seed
AT5G27880	C2H2		6.20	6.63	0.001	C	EN
AT1G60280	NAC	NAC23	4.40	4.29	0.003	S,(E)	ne
AT5G54070	HSF	HSFA9	4.46	4.25	0.002		
AT5G11050	MYB	MYB64	6.24	4.18	0.001	C,(E),(S)	EN
AT2G40220	AP2-EREBP	ABI4	3.77	3.93	0.042	E	EM
AT2G24840	MADS	AGL61	7.73	3.90	0.002		
AT5G41090	NAC	NAC95	4.88	3.81	0.002	A	ne
AT1G55600	WRKY	MINI3/WRKY10	1.89	3.75	0.006	ne	EN^4^
AT1G66390	MYB	MYB90	1.77	3.68	0.071		
AT5G61890	AP2-EREBP		1.84	3.07	0.014		
AT1G21970	CCAAT-HAP3	LEC1	4.26	3.03	0.063		
AT5G01860	C2H2		3.60	2.88	0.002	A,C,(E),(S)	(EN)
AT4G00540	MYB	MYB3R2	1.81	2.88	0.019		
AT1G01530	MADS	AGL28	3.40	2.84	0.013		
AT5G56200	C2H2		5.18	2.62	0.014	A	EN^4^
AT1G35520	ARF	ARF15	1.59	2.55	0.012	A	ne
AT1G67030	C2H2	ZFP6	3.73	2.43	0.029	A	ne
AT5G45980	Homeobox	WOX8	4.51	2.30	0.006	E	EM
AT1G56650	MYB	MYB75	1.89	2.26	0.081		
AT5G58850	MYB	MYB119	7.77	2.23	0.017		
AT4G38070	bHLH		2.13	2.22	0.020		
AT1G75250	MYB-related	RL6	3.02	1.71	0.011	C,E,S	EN
AT2G33710	AP2-EREBP		1.85	1.61	0.002	E,(S)	EM,EN
AT1G49770	bHLH	ZOU/RGE1/bHLH95	2.09	1.61	0.063	C,(S)	EN
AT3G01030	C2H2		3.81	1.43	0.010	S,(C)	ne
AT1G04370	AP2-EREBP	ERF14	3.24	1.11	0.096		
AT2G22750	bHLH		2.46	0.80	nd	(A)^5^	ne
AT5G01380	Trihelix		1.57	0.06	nd	A	ne
AT5G50490	CCAAT-HAP5		2.11	-0.27	nd	C	EN

The genes are ranked in descending order based on the average ΔΔC_T _value in the secondary qRT-PCR screen. Average ΔΔC_T _values and *P *values (Student's *t*-test) were calculated as described in Methods. GFP activity in female gametophytes was analyzed at one day after emasculation (corresponding to the mature stage of female gametophyte development, FG7 [63]) and in early-developing seeds at flower stage 16 (corresponding to endosperm stages V to VI and embryo stages from elongated zygote to two-cell embryo-proper [68,69]). A, antipodal cells; C, central cell; E, egg cell; EM, embryo; EN, endosperm; ne, no consistent expression; nd, not determined; S, synergid cells; (), weak expression.

^1 ^Arabidopsis Genome Initiative number.

^2 ^Based on the classification of Arabidopsis transcription-factor genes provided by AGRIS http://arabidopsis.med.ohio-state.edu/AtTFDB and DATF http://datf.cbi.pku.edu.cn.

^3 ^Based on gene annotation provided by TAIR http://www.arabidopsis.org.

^4 ^GFP activity was more intense in the micropylar endosperm compared to the chalazal and peripheral endosperm.

^5 ^GFP activity was not detected at FG7 but was detected at FG5.

The cells of the female gametophyte are in close proximity especially in the micropylar pole where the egg cell is flanked by the two synergid cells and is positioned adjacent to the central cell cytoplasm [63]. Using epifluorescence microscopy, it proved difficult to unambiguously determine the cellular patterns of promoter activity for genes that showed multi-cell-type expression patterns (Figures 1C, D and 2D). In addition, our qRT-PCR analysis (Additional file 3) suggests that most of the transcription-factor genes are expressed at low levels, which would produce weak GFP signals in *promoter:GFP *analyses. Therefore, we generated a nuclear-localized version of GFP by fusing the coding region of an Arabidopsis histone *H2B *gene (*HTB2*, *AT5G22880*) to the N-terminus of a single copy of *GFP *(*n1GFP*) or to two tandemly fused copies of *GFP *(*n2GFP*) in order to increase the resolution and sensitivity of our *promoter:GFP *analysis.

To test the utility of the nuclear-localized GFP constructs during female gametophyte development, we generated promoter constructs for genes *AT5G27880 *and *AT5G01860 *each fused to the *n1GFP *and *n2GFP *reporter genes, and compared their expression patterns in the mature female gametophyte to those obtained with the cGFP reporter (Figure 2). Activities of both n1GFP and n2GFP driven by the *AT5G27880 *promoter were localized in the central cell nucleus (*pAT5G27880:n1GFP *and *pAT5G27880:n2GFP*; Figure 2B, C). In rare instances, weak antipodal expression was also observed (Additional files 5 and 6). This expression pattern agreed with the pattern obtained with the cGFP construct (*pAT5G27880:GFP; *Figure 2A). For the *pAT5G01860:n1GFP *and *n2GFP *fusions, strong GFP activity was detected in the central cell and antipodal cell nuclei, and weaker activity was detected in the egg cell and synergid cell nuclei (Figure 2E, F). The expression patterns obtained for the *n1GFP/n2GFP *constructs were similar to that of the *cGFP *construct except that the cGFP antipodal signals were generally weaker and only observed in rare instances (Figure 2D-F, Additional file 6). We did not detect any adverse effects of *n1GFP *or *n2GFP *expression on female gametophyte development or function (data not shown); this observation is in agreement with previous reports where histone H2B fusions with yellow fluorescent protein or GFP were used in both animal and plant model systems without any detrimental effects on viability or development [69,70]. Moreover, we did not find any qualitative differences in the patterns of expression for *n1GFP *versus *n2GFP *constructs for the same promoter sequences (Figure 2B, C, E, F). These results demonstrate that the use of n1GFP/n2GFP reporters improved the sensitivity and spatial resolution of *promoter:GFP *analysis for studying gene expression patterns during female gametophyte development.

**Figure 3:**
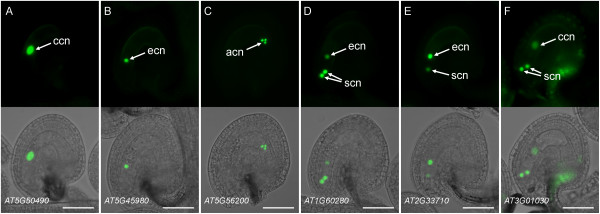
**Expression patterns of transcription-factor *promoter:n1GFP/n2GFP *fusions in the mature female gametophyte**. (A) *pAT5G50490:n1GFP *expression in the central cell. (B) *pAT5G45980:n1GFP *(*WOX8*) expression in the egg cell. (C) *pAT5G56200:n1GFP *expression in the antipodal cells. (D) *pAT1G60280*:*n1*GFP (*NAC23*) expression primarily in the synergid cells with weaker activity in the egg cell. (E) *pAT2G33710:n1GFP *expression primarily in the egg cell with weaker activity in the synergid cell. (F) *pAT3G01030:n2GFP *expression primarily in the synergid cells with weaker activity in the central cell and the sporophytic cells of the integument and funiculus. Each panel contains an epifluorescence image (top) and an overlay (bottom) of the epifluorescence and a bright-field image of the same ovule. acn, antipodal cell nuclei; ccn, central cell nucleus; ecn, egg cell nucleus; scn, synergid cell nuclei. Scale bars: 50 μm.

We constructed promoter fusions for 18 additional genes using *n1GFP *(15 genes) or *n2GFP *(3 genes) reporter constructs (Additional file 5). Eleven promoter fusions showed diverse patterns of expression in the mature female gametophyte (Table 1, Figure 3, Additional file 5), while the remaining seven genes did not show any reproducible level of GFP activity in mature female gametophytes (Additional file 5). However, all of the latter genes showed specific expression in the seed or in the sporophytic tissues of the ovary (Additional files 5 and 7) indicating that the constructs were functional. The *promoter:n1/n2GFP *constructs representing 13 genes (including the two described above) exhibited consistent patterns of GFP activity in the majority of primary transgenic (T1) lines (Figures 2 and 3, Additional file 5). As shown in Figures 2 and 3, expression in one, two, or all four cell types of the female gametophyte was detected using the *n1/n2GFP *constructs. Single-cell-type expression in the central cell, egg cell and antipodal cells was detected for nine genes including *AT5G50490 *(Figure 3A), *AT5G45980/WOX8 *(Figure 3B), and *AT5G56200 *(Figure 3C), respectively. Three promoter-fusion constructs showed expression in two cell types (usually one cell type with a higher level of expression): synergid-egg cells (*AT1G60280/NAC23 *and *AT2G33710*; Figure 3D, E) and synergid-central cells (*AT3G01030*; Figure 3F). Finally, expression of the *AT5G01860 *promoter-fusion was detected in all four cell types of the female gametophyte (Figure 2E, F). Some of the constructs showed a secondary set of expression patterns in a minority of T1 lines (Additional files 5 and 6); these patterns may be attributable to the sensitivity of the *n1/n2GFP *reporter constructs which potentially report low levels of endogenous gene transcription not detected using previous approaches. For all reporter constructs, n1/n2GFP localization was nuclear (Figures 2 and 3, Additional file 6) except for *AT5G41090/NAC95 *where both nuclear and cytosolic localization patterns were observed (Additional file 6). Cytosolic localization may have resulted from an excessive amount of n2GFP fusion protein produced by a highly active promoter as the C_T _value for *NAC95 *in *ms1 *ovaries (26.32 ± 0.27, mean ± s.d.) was the lowest (suggesting high mRNA prevalence) among the 20 transcription-factor genes analyzed using *n1/n2GFP *promoter-fusion constructs (Additional file 3). Taken together, these expression patterns demonstrate that we have identified 17 transcription-factor genes that are transcribed during late stages of female gametophyte development in Arabidopsis (Table 1, Additional file 5).

### Patterns of transcription-factor gene promoter activities during megagametogenesis and early seed development

**Figure 4:**
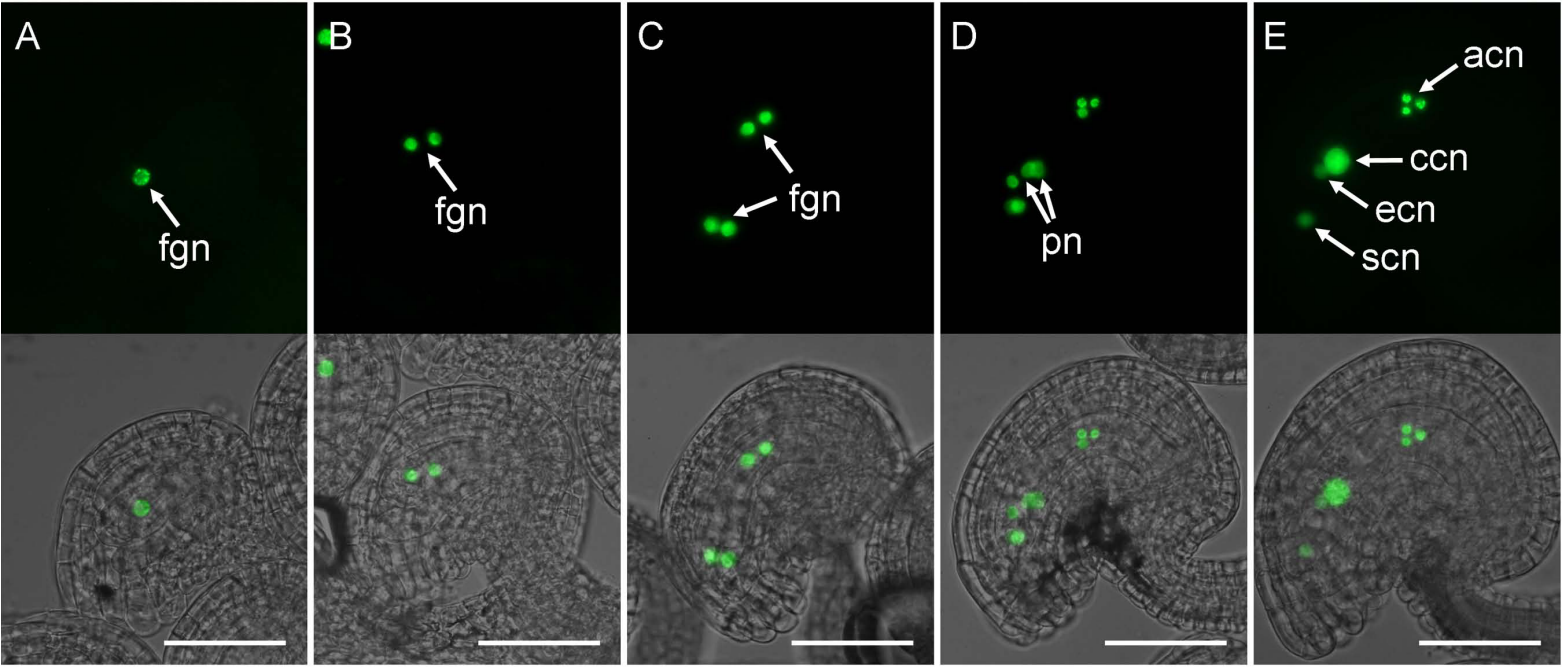
**Expression of *pAT5G01860:n1GFP *during megagametogenesis**. Expression at developmental stage FG1 (A), FG2 (B), FG4 (C), FG5 (D), and FG6 (E) of the developing female gametophyte [63]. Each panel contains an epifluorescence image (top) and an overlay (bottom) of the epifluorescence and a bright-field image of the same ovule. acn, antipodal cell nuclei; ccn, central cell nucleus; ecn, egg cell nucleus; fgn, female gametophyte nuclei; pn, polar nuclei; scn, synergid cell nucleus. Scale bars: 50 μm.

Megagametogenesis and early endosperm development in Arabidopsis are characterized by a series of nuclear divisions followed by cellularization [1,2,71]. We first examined the expression patterns of our reporter constructs at developmental stages FG4 to FG5 of megagametogenesis before the mature, cellularized stage of female gametophyte development. Of the 19 genes tested, we did not detect a high level of promoter activity at these stages for most of the genes tested (data not shown) except for *AT5G01860*. As shown in Figure 4, the promoter activity for *AT5G01860 *was observed in all nuclei from developmental stages FG1 to FG5 [63] with equal signal intensity. After the fusion of polar nuclei, the level of GFP activity increased in the central cell and the antipodal cells as compared to the egg cell and the synergid cells (Figure 4E). In this case, our data indicate that the initial transcriptional activity of *AT5G01860 *occurs as early as the initiation of megagametogenesis and that modulation of expression in late female gametophyte stages occurs after polar-nuclear fusion and immediately prior to fertilization.

**Figure 5:**
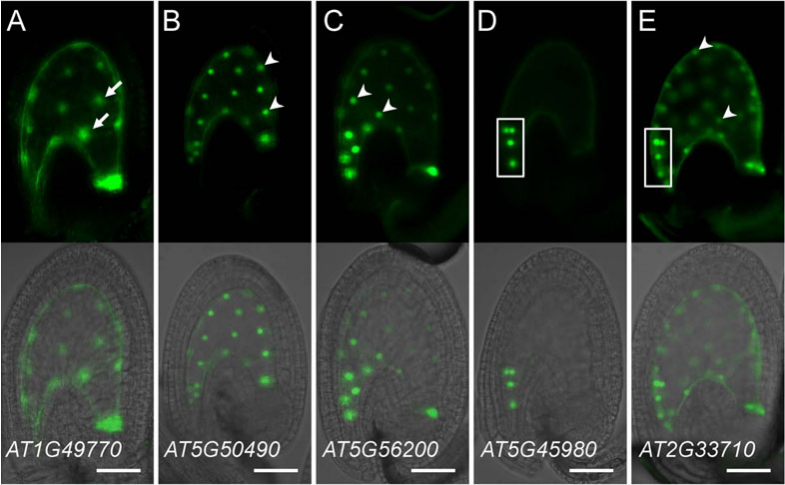
**Expression patterns of transcription-factor *promoter:cGFP *and *promoter:n1GFP *fusions during early seed development**.(A) *pAT1G49770:cGFP *(*ZOU/RGE1*) activity in endosperm nuclear cytoplasmic domains (NCDs) [104]. (B) *pAT5G50490:n1GFP *activity in endosperm nuclei. (C) *pAT5G56200:n1GFP *activity primarily in micropylar endosperm nuclei. (D) *pAT5G45980:n1GFP *(*WOX8*) expression in pro-embryo nuclei. (E) *pAT2G33710:n1GFP *activity in both endosperm and pro-embryo nuclei. Each panel contains an epifluorescence image (top) and an overlay (bottom) of the epifluorescence and a bright-field image of the same seed. The micropylar region of the seed is oriented towards the left side of the panel. Arrows point to endosperm NCDs, and arrowheads point to endosperm nuclei. Boxed areas indicate the micropylar location of the embryos. Scale bars: 50 μm.

To determine if the female gametophyte-expressed transcription-factor genes are also expressed during endosperm and/or embryo development, we analyzed all of our reporter constructs at flower stage 16 [68], which corresponds to stages V (16-nucleate stage) to VI (~30-nucleate stage) of endosperm development and in the elongated zygote to two-cell embryo-proper stage of embryogenesis [68,69]. We observed consistent embryo and/or endosperm expression for 12 promoter-fusion constructs (Additional file 5). The majority of the reporter constructs (7 genes; Additional file 5) showed a relatively uniform pattern of expression in the endosperm when assayed using cGFP (*ZOU*; Figure 5A) or nuclear-localized GFP (*AT5G50490*; Figure 5B) while reporter constructs for genes *AT1G55600*/*MINI3 *and *AT5G56200 *showed preferential GFP activity in the micropylar endosperm (Figure 5C, Additional file 5). With two genes, *WOX8 *and *AT2G40220*/*ABI4*, expression was observed in early embryos with very low or no detectable activity elsewhere in early seeds (Figure 5D, Additional file 5). Finally, the *AT2G33710 *promoter-fusion construct showed expression in both the embryo and endosperm (Figure 5E). Of the 17 transcription-factor genes expressed in the female gametophyte, 10 showed consistent expression in the seed (Table 1). Together, our analysis indicates that a significant portion of the female gametophyte-expressed transcription-factor genes are also transcribed during early seed development.

## Discussion

### Utility of our qRT-PCR-based differential-expression screen

We performed a two-step qRT-PCR screen to identify transcription-factor genes with reduced mRNA levels in *dif1 *ovaries compared to *ms1 *ovaries. In the primary screen, 69 genes were identified as *dif1 *down-regulated (reduced levels in *dif1 *as compared to *ms1*) while 58 genes were identified as *dif1 *up-regulated (Additional files 1 and 2). Although *dif1 *and *ms1 *ovules are apparently indistinguishable in terms of integument morphology [56], the other sporophytic organs/tissues of the *dif1 *ovaries may show altered gene expression patterns due to the absence of the female gametophyte. Therefore, in addition to the female gametophyte-expressed genes that are of primary interest to this study, the *dif1*-down-regulated genes might include sporophyte-expressed genes that are induced by the female gametophyte. While this is an unlikely possibility for all of the transcription-factor genes assayed, the promoter-fusion assays were designed, in part, to address this issue (Additional file 4). On the other hand, the *dif1*-up-regulated genes may include genes that are normally repressed in the ovary by the female gametophyte. Although sporophyte-expressed genes of this type are not the focus of this study, these transcription-factor genes, once confirmed, could represent important developmental regulators mediating the communication between the female gametophyte and the surrounding sporophytic organs and tissues.

Of the 69 *dif1*-down-regulated genes, 26 genes were further validated in an additional series of qRT-PCR assays (secondary screen) under more stringent PCR conditions (Additional file 3). We and others previously used microarray-based differential expression screens to identify a total of 63 unique transcription-factor genes with reduced mRNA levels in the ovules or pistils of *spl*, *coa *or *dif1 *mutant plants (Additional file 8) [54,56,57]. An additional screen comparing *dif1 *ovules to fertilized wild-type seeds led to the identification of another five transcription-factor genes (Additional file 8) [55]. However, there is no significant overlap in the detection of genes among the published microarray-based screens and the present study (Additional file 8). This discrepancy may be attributed, in part, to differences in sampling of the material (e.g., ovules versus ovaries), the developmental staging of the material (e.g., mature versus developing female gametophytes), or any effect the mutations might have had on the sporophytic tissues of the sampled material (i.e., ovule, ovary, seeds or pistil). A direct comparison of the approaches awaits further confirmation of the expression of the candidate genes.

Of the 24 novel genes identified in this study (Additional files 3 and 8) [54-57], 13 genes were analyzed using promoter fusions with 12 showing transcriptional activity in the female gametophyte (Table 1). Such a high proportion of validated genes suggests that the majority of the original 24 genes uniquely identified in our qRT-PCR screen are likely transcribed in the female gametophyte. The failure to identify these 24 genes in the previous studies could be due to low mRNA levels that are below the detection limits of the microarray assays. This conclusion is supported by our observation that the majority of these 24 genes showed relatively high C_T _values (low mRNA levels) in *ms1 *ovaries: 22 genes displayed normalized C_T _values above 28 (Additional file 3), while the two genes (*MYB64 *and *NAC95*) identified in the previous microarray studies displayed C_T _values between 26 and 28 (Additional file 3). Taken together, our results demonstrate that our qRT-PCR-based differential expression screen is sensitive enough to detect low-prevalence mRNAs of transcription-factor genes in the female gametophyte.

### Identification of transcription-factor genes expressed in the female gametophyte

We tested 15 of the 26 transcription-factor genes exhibiting reduced mRNA levels in *dif1 *ovaries in our qRT-PCR-based screen (Additional file 3) using *promoter:GFP *fusions and confirmed that 14 are transcribed within the female gametophyte (Table 1, Figures 1, 2, and 3). The single non-expressing-promoter construct (*MINI3*) may not have contained all the *cis*-regulatory sequences required for proper expression in the female gametophyte. Of the 11 genes that were not tested using promoter fusions, one (*AGL61*) had been isolated using a similar differential screen and its promoter activity in the female gametophyte had been described previously [7,9]. Therefore, it is likely that most of the remaining untested genes are also expressed in the female gametophyte. In addition, we tested nine genes below the statistical threshold of the secondary screen using promoter fusions (Additional file 5). Three exhibited female gametophyte expression (Table 1, Figures 1A and 3A), suggesting that additional female gametophyte-expressed genes can be identified in this group. Of a total of 17 genes exhibiting female gametophyte expression, 10 were predominantly transcribed in a single cell type: the egg cell (2 genes), the central cell (2 genes), or the antipodal cells (6 genes; Table 1). The remaining seven genes were transcribed in multiple cell types within the female gametophyte (Table 1).

Our analyses uncovered novel patterns of transcription-factor gene activity in the female gametophyte and during early seed development. We also confirmed some previously described patterns of gene activity, supporting our general approach to identifying low-prevalence transcription-factor mRNAs in the female gametophyte. Six transcription-factor genes displayed strong *promoter:GFP *activity exclusively (*AT5G50490 *and *AT5G27880*) or predominantly (*ZOU*/*RGE1*, *RL6*, *AT5G01860*, and *MYB64*) in the central cell and also in the endosperm (Table 1; Figures 1B-D, 2, 3A and 5A, B). Among these, *ZOU/RGE1/bHLH95 *encodes a bHLH transcription factor that has been proposed to regulate endosperm adhesion and breakdown which, in turn, is required for the proper epidermal development of the embryo [72,73]. Our promoter-fusion analysis suggests that *ZOU/RGE1 *is transcribed in the central cell, the synergid cells, and the endosperm (Table 1; Figures 1B and 5A). This differs from the previous reports where expression of *ZOU/RGE1 *was only observed in the endosperm [72,73]. The discrepancy may be due to different promoter lengths and the type of reporter genes used in the studies. *RL6 *encodes a MYB-related transcription factor expressed in the micropylar endosperm [74]. The expression patterns of the remaining four "central cell-endosperm" genes (*AT5G01860*, *AT5G27880*, *AT5G50490*, and *MYB64*) have not been described previously. However, supporting our promoter-reporter data, an analysis of the publicly available microarray data using Genevestigator indicates mRNA accumulation for *AT5G01860 *and *MYB64 *in the endosperm https://www.genevestigator.com.

In contrast to the genes with transcriptional activity in both the central cell and the endosperm, we detected endosperm expression for three genes that showed no expression in the central cell (Table 1, Figure 5C, E). The transcriptional activity of *AT5G56200 *or *AT2G33710 *has not been described previously. However, available microarray data indicate mRNA accumulation for both genes in laser-captured endosperm samples https://www.genevestigator.com[75]. *MINI3 *encodes a WRKY transcription factor that has been shown to regulate endosperm growth and cellularization [76]. We detected *MINI3 *promoter activity in the endosperm but not in the female gametophyte (Table 1) in agreement with a previous report [76].

Three transcription-factor genes exhibited strong *promoter:GFP *activity exclusively (*ABI4 *and *WOX8*) or predominantly (*AT2G33710*) in the egg cell (Table 1, Figure 3B, E). All three were expressed in the embryo after fertilization (Table 1, Figure 5D, E). *ABI4 *is primarily expressed in the embryo during seed maturation and encodes an AP2-EREBP transcription factor required for ABA and sugar signaling during seed development and germination [77-79]. Transcription of *ABI4 *in the egg cell has not been reported previously. *WOX8 *encodes a homeobox transcription factor that is involved in regulating early embryo development [80-82]. Using *in situ *hybridization, *WOX8 *mRNA has been localized to the egg cell and the basal-cell lineage of the developing embryo [80]. However, our *WOX8 *promoter fusion showed expression in both the apical and basal cell lineages of the embryo (Figure 5D). This construct may not contain all of the *cis*-regulatory elements required for proper transcription of the reporter gene including elements to suppress transcription in the apical cell lineage. Alternatively, WOX8 mRNA may be degraded preferentially in the apical-cell lineage or be redistributed to the basal cell upon the first zygotic division as has been suggested previously [80]. We also detected reduced mRNA levels in *dif1 *ovaries for *AT5G54070*/*HSFA9 *and *AT1G21970*/*LEC1 *(Table 1) previously shown to be required for embryogenesis [83,84]. Transcriptional patterns of these two genes in the female gametophyte remain to be determined.

We found that among the 17 transcription-factor genes active in the female gametophyte, nine genes are highly transcribed in the synergid or the antipodal cells (Table 1), the two cell types of the female gametophyte that do not give rise to any cells or tissues of the developing seed. The promoter fusions for *NAC23 *and *AT3G01030 *were expressed predominantly in the synergid cells with weak expression in the egg cell or the central cell (Table 1). This is essentially similar to the expression pattern observed for *MYB98 *[22,85]. We found that *RL6 *is also expressed in the synergid cells, the central cell, and the egg cell (Table 1). *MYB98 *has been shown to control at least a portion of the gene regulatory network required for proper synergid cell differentiation [26]. Therefore, *NAC23*, *AT3G01030 *and *RL6 *may constitute additional components of the same or a larger network controlling synergid cell differentiation and function. At the opposite, chalazal pole, we have identified six transcription-factor genes that are highly expressed in the antipodal cells (Table 1, Figures 2E, F and 3C). The function of these genes is not known. Among them, *AT1G35520*/*ARF15 *might play a role in the transcriptional regulatory networks responding to the plant hormone auxin, which has been shown recently to regulate cell fate specification in the female gametophyte [86].

Among the 26 transcription-factor genes we have described here (Additional file 3), only one gene (*AGL61*) previously had been shown to be functionally required in the female gametophyte [7,9]. It is possible that gene redundancies or functional compensations may have precluded isolation of these transcription-factor genes in genetic screens for female-gametophyte mutants. This hypothesis is supported by the observation that some of the genes identified in this study are structurally similar. For example, three (*AT1G67030*, *AT5G01860*, and *AT5G27880*) of the five C2H2-type transcription-factor genes we identified are closely related homologues [87]. Interestingly, a number of the female gametophyte-expressed transcription-factor genes isolated in this study (see above) have been shown to be required after fertilization [72,73,76-84,88-91]. It remains to be determined whether they perform any function in the female gametophyte as individual genes, or as members of multi-gene families with complex expression programs encompassing both the gametophytic and the zygotic portions of the plant life cycle.

## Conclusions

To begin to identify the gene-regulatory networks controlling angiosperm female gametophyte development, we performed a large-scale qRT-PCR-based differential expression screen of nearly all transcription-factor genes in Arabidopsis ovaries. Compared to microarray-based methods [54-57], our approach proved to be more sensitive and allowed the identification of 26 transcription factor genes, the majority of which previously had not been identified as female gametophyte-expressed genes. We further confirmed transcriptional activity of 17 genes in the female gametophyte using promoter-fusion analyses. Using a nuclear-localized GFP reporter, we have developed female gametophyte-cell-specific marker lines well suited for monitoring individual cell fates during female gametophyte development and early seed development. Transcription factors are at the center of gene regulatory networks and perform essential regulatory roles in development and cell differentiation [33]. The transcription-factor genes identified in this study can be used to dissect the gene regulatory networks in the female gametophyte through a combination of genetic and molecular approaches, ultimately enabling a better understanding of the molecular mechanisms that control female gametophyte development and function during plant reproduction.

## Methods

### Plant material and growth conditions

The *dif1-2 *and *ms1-1 *mutant alleles, initially isolated in the Landsberg *erecta *(L*er*) background [60,67], were backcrossed four and seven times, respectively, to L*er*. Plant transformations were performed with the Columbia-0 (Col-0) ecotype. Arabidopsis plants were grown at 22°C under continuous light or 16-hr light/8-hr dark photoperiod as described previously [19,56].

### Tissue collection, RNA isolation and cDNA synthesis

Homozygous *dif1 *and *ms1 *mutant plants were identified based on their male-sterile phenotype among the progeny from a self-fertilization of *dif1/+ *and *ms1/+ *parents [60,66,67,92]. Ovaries were collected from *dif1 *and *ms1 *mutant plants by removing the stigma and style from the pistils harvested at flower stages 12C [63] to 13 or 14 [68]. Seven pairs of *dif1 *and *ms1 *RNA samples were used in this study (Additional file 9). Each RNA sample was extracted from 100 to 150 ovaries using the RNeasy^® ^Plant Mini Kit (Qiagen, Hilden, Germany) or TRIzol^® ^Reagent (Invitrogen, Carlsbad, USA) and purified with the RNeasy^® ^MinElute Cleanup Kit (Qiagen) after a treatment with TURBO DNase (Ambion, Austin, USA) to remove any genomic DNA. The purified RNAs were then reverse transcribed with Oligo-dT primers using the RETROscript^® ^Kit (Ambion) or SuperScript III Reverse Transcriptase (Invitrogen) following the manufacturer's instructions. For most samples, first-strand cDNA was further purified using the MinElute^® ^PCR Purification Kit (Qiagen) after an RNase H treatment (New England Biolabs, Beverly, USA).

### Primer design

The majority of the primer-pairs (1,356 out of 1,482) used in the primary qRT-PCR screen (Additional file 1) were described previously [93]. The rest of the primer-pairs were designed *de novo*, including primers for 70 additional transcription-factor genes annotated by the Arabidopsis Gene Regulatory Information Server http://arabidopsis.med.ohio-state.edu[94,95] or the Database of Arabidopsis Transcription Factors http://datf.cbi.pku.edu.cn[96]. The same primers were used in the secondary qRT-PCR screen with the exception of 17 primer-pairs, which were redesigned to ensure specific PCR amplification (Additional file 3). The primer sequences were designed based on gene structure models at the Arabidopsis Information Resource http://www.arabidopsis.org (TAIR) using LightCycler Probe Design Software 2.0 (Roche, Mannheim, Germany) and by setting the primer melting temperature at 60°C. The amplicon sizes ranged between 68 and 261 bp. The transcription-factor genes analyzed in this study and the corresponding primer-pair sequences are listed in Additional files 1 and 3. Based on analysis of melting curves (see below), 94.7% of the primer pairs produced a single identical PCR product for each pair of *ms1*-*dif1 *qRT-PCR reactions, indicating that a significant majority of the primer pairs were gene specific under our experimental conditions (Additional file 1).

### Quantitative RT-PCR experimental setup and data analysis

Quantitative RT-PCR was performed using a LightCycler 1.5 instrument in a 32-capillary format (Roche). The PCR program for the primary screen consisted of an initial denaturing step at 95°C for 5 min, followed by 45 cycles at 95°C for 15 s, 60°C for 15 s, and 72°C for 10 s. The cycle parameters were changed to 95°C for 10 s, 60°C for 5 s, and 72°C for 10 s in the secondary screen in order to increase the stringency of the PCR reactions. Standard melting-curve analysis provided by the instrument manufacturer was performed after each PCR run to determine whether a single PCR product was amplified in each reaction and whether the same product was amplified from both *ms1 *and *dif1 *cDNA sources. The C_T _values were calculated using the standard approach provided in the LightCycler software 4.0 package (Roche).

To determine whether *ACT2 *is a suitable reference gene, levels of *ACT2 *mRNA were quantified with qRT-PCR in three independent pairs of *ms1 *and *dif1 *RNA samples. All experimental procedures from reverse transcription to qRT-PCR analysis were conducted side-by-side using the same amount of RNA. The C_T _values for *ACT2 *in *ms1 *(18.39 ± 0.29, mean ± s.d.) and *dif1 *(18.40 ± 0.17, mean ± s.d.) RNAs did not differ significantly (*P *= 0.93, paired Student's *t*-test), indicating that the level of *ACT2 *mRNA in the ovary is not affected by the absence of the female gametophyte.

Each qRT-PCR run consisted of 16 pairs (*dif1 *and *ms1*) of 10-μl reactions containing 2.5 or 5.0 pmol of primers and an aliquot of the master mix, which was assembled by combining the *dif1 *or *ms1 *cDNA with the master mix from the LightCycler^® ^FastStart DNA Master^PLUS ^SYBR Green I Kit according to manufacturer's instructions (Roche). The amount of cDNA used in the master mix was adjusted so that each reaction contained cDNA from 10-15 ng of total RNA. To account for the variation in template concentration between PCR runs, each run contained a pair of *ms1*- *dif1 *reactions for *ACT2*, which was used to normalize the C_T _values of the target genes analyzed in the same run (normalized C_T, target _= C_T, target _- C_T, *ACT2 *_+19). The majority of the qRT-PCR runs produced C_T _values around 19 for *ACT2*. The amount of cDNA template for a specific transcription-factor gene was considered negligible when the C_T _value was at or above 36. Therefore, the normalized C_T _values were manually cut off at 36. Differences in mRNA levels between *ms1 *and *dif1 *RNA samples were calculated using the -ΔΔC_T _method [97] where ΔΔC_T _= normalized C_T, *dif1 *_- normalized C_T, *ms1*_.

We have previously shown that qRT-PCR is sensitive enough to detect reduced *MYB98 *mRNA level in *dif1 *versus wild-type pistils [22]. Therefore, a pair of *ms1*- *dif1 *reactions for *MYB98 *was included in each PCR run as a positive control. All seven pairs of *ms1*- *dif1 *RNA samples used in this study exhibited significantly higher levels of *MYB98 *mRNA in ovaries from *ms1 *as compared to *dif1 *mutant plants (*P*< 2.50E-05, Student's *t*-test; Additional file 9).

In the primary screen, the majority of genes (1,265 out of 1,482) were analyzed once, and the ΔΔC_T _was calculated as described above. Additional replicates using either the same or different RNA samples were conducted for the remaining 217 genes, and an average ΔΔC_T _was calculated by averaging data from technical replicates followed by averaging the resulting means for biological replicates. Calculation of the Pearson correlation coefficients and the Student's *t*-test were performed using the corresponding functions in Excel (Microsoft, Redmond, USA).

### Promoter-fusion constructs

Four binary transformation vectors (pBI-GFP[S65T] [98], pBI-1GFPB, pBI-n1GFP, and pBI-n2GFP) were used in the construction of promoter fusions with reporter genes *GFP*, *n1GFP*, and *n2GFP *(Additional file 10); these vectors were generated as follows. A synthetic linker containing multiple cloning sites (5'- aagcttcctgcaggttaacagtactcacgtgaggcctactagtgagctcggtaccctcgaggtcgactctagaggatcc-3') was cloned into the pBI-GFP[S65T] vector [98] between *Hind*III and *Bam*HI sites resulting in pBI-1GFP.

The GFP coding region was amplified from pBI-GFP[S65T] [98] with the forward primer (5'-caaacaacgggatccatggtgagcaagggcgag-3') and the reverse primer encoding an (Ala)_8 _linker (5'-aaaaaaagaagatctagcagcagcagcagcagcagcagccttgtacagctcgtccatgc-3'). The PCR product was then digested with *Bam*HI and *Bgl*II and inserted into the *Bam*HI (compatible with *Bgl*II) site of pBI-1GFP resulting in pBI-2GFP.

Two DNA oligonucleotides (5'-tcgactctagaggatccggccggcctggaggtggaggtggagcta-3' and 5'-gatctagctccacctccacctccaggccggccggatcctctagag-3') were annealed to generate a linker with *Sal*I/*Bgl*II (5'/3') cohesive ends, which contained an *Xba*I and a *Bam*HI site upstream of sequences encoding the peptide GlyArgPro(Gly)_5_Ala. This linker was then cloned into pBI-1GFP between *Sal*I and *Bam*HI sites resulting in pBI-1GFPB.

The coding region of the *HTB2 *gene was amplified from Col-0 genomic DNA with the forward primer HTB2F (5'-gactcgggatccatggcgaaggcagataagaaacc-3') and the reverse primer HTB2R2 encoding an (Ala)_4 _linker (5'-gaaaaaaagaagatctagcagcagcagcagaactcgtaaacttcgtaaccgc-3'). Two silent mutations (ATC_60 _to ATA and CTT_101 _to CTG) were introduced via a series of PCR reactions to remove *Hind*III and *Bgl*II sites from the coding region of *HTB2*. To do this, the coding region of *HTB2 *was amplified as two separate fragments using primer pairs HTB2F plus HTB2LUR2 (5'- cttgcttcaacaccttgaagatgtaTatcttgtatgtctcaacgttcttc -3', the mutagenized nucleotide is uppercase), and HTB2LDF2 (5'- gaagaacgttgagacatacaagatAtacatcttcaaggtgttgaagcaag -3') plus HTB2R2. The two fragments were then fused through an overlap-extension PCR [99,100] using the primer pair HTB2F plus HTB2R2 resulting in a full-length *HTB2 *PCR product containing the mutation ATC_60 _to ATA. This product was further mutagenized by repeating the steps described above with a different set of primers: HTB2F plus HTB2LUR (5'- ggcttcttgttgtacctcgcCagcttcgaagactcaccagc -3') and HTB2LDF (5'- gctggtgagtcttcgaagctGgcgaggtacaacaagaagcc -3') plus HTB2R2. The resulting PCR product containing both mutations was digested with *Bam*HI and *Bgl*II and inserted into the *Bam*HI site of pBI-1GFP and pBI-2GFP resulting in pBI-n1GFP and pBI-n2GFP, respectively.

For each transcription-factor gene analyzed, promoter regions containing between 800 and 2,700 bp of the upstream and between 9 and 39 bp of the downstream sequence to the translation start site were amplified from Col-0 genomic DNA using primers containing restriction sites or homologous recombination sites (Additional file 10). The PCR products were inserted into pBI-GFP[S65T] [98], pBI-1GFPB, pBI-n1GFP, or pBI-n2GFP using conventional cloning procedures based on restriction digestion or through the use of the In-Fusion technology according to manufacturer's instructions (BD Biosciences, Heidelberg, Germany) as indicated in Additional file 10. The PCR amplifications were performed using PfuUltra (Stratagene, La Jolla, USA) or ExTaq (Takara, Otsu, Japan) DNA polymerases. All promoter-vector junctions were verified by sequencing.

### Plant transformation

Arabidopsis plants were transformed as described previously [19,56] with *Agrobacterium tumefaciens *strains GV3101 pMP90 [101] or LBA4404 [102] containing the GFP binary vectors using the standard floral dip method [103]. The presence of the transgene in each T1 plant was confirmed using PCR.

### Analysis of gene-promoter activity

GFP activity within the mature female gametophyte was analyzed one day after emasculation as previously described [56]. Expression patterns during early female gametophyte development were analyzed in ovules from floral stages 12B to 13 [63,68] containing female gametophytes at developmental stages FG1 to FG6 [63]. Seeds were analyzed in self-fertilized siliques at floral stage 16 [68] containing early nuclear stages of endosperm development [69]. Promoter activity within the mature female gametophyte was analyzed in T1- or T2-generation plants. Between 5 and 16 T1 lines per construct (Additional file 5) were examined to verify the expression patterns described above. Transformation of plants with promoter-less pBI-n1GFP or pBI-n2GFP vectors did not produce any detectable levels of GFP activity above background in the relevant tissues.

Image acquisition and processing were carried out as described previously [19,56]. In brief, the bright-field and epifluorescence images of ovules and young seeds were captured using either a MicroFire CCD camera (Optronics, Goleta, USA) or an AxioCam MRm REV2 camera (Carl Zeiss, Jena, Germany) attached to an Axiophot or an Axioplan compound epifluorescence microscope (Carl Zeiss) equipped with an enhanced GFP bandpass filter (filter set 38 HE EGFP, exciter 450-490 nm, dichroic 495 nm, emitter 500-550 nm; Carl Zeiss). Image processing, including creation of the overlays of epifluorescence and bright-field images, was performed using Photoshop CS (Adobe Systems Inc., San Jose, USA).

## Authors' contributions

GND, RY and KSS conceived the research, supervised and coordinated all research activities. DW, CZ, DJH, I-HK and JAP carried out qRT-PCR reactions. CZ and DJH generated transcription factor promoter constructs. CZ, DW and MIS generated and analyzed transgenic lines. DW, RY, KSS and GND analyzed all data and wrote the manuscript. All authors read and approved the final manuscript.

## Supplementary Material

Additional file 1 **Primary qRT-PCR screen of Arabidopsis transcription-factor genes**. Provided information includes: AGI numbers, primer sequences, amplicon sizes, and C_T _values for 1,482 transcription-factor genes analyzed in the primary qRT-PCR screen. Click here for file

Additional file 2 **Summary of the primary qRT-PCR screen**. (A) Comparison of mRNA levels in *ms1 *and *dif1 *ovaries. Average C_T _values for the *ms1 *and *dif1 *ovary RNAs were compared for each transcription-factor gene analyzed. Dashed lines indicate the threshold of the primary screen (ΔΔC_T _> 1.5 or ΔΔC_T _< -1.5). (B) Histogram illustrating the distribution of ΔΔC_T _values obtained for all transcription-factor genes in the primary screen. Click here for file

Additional file 3 **Secondary qRT-PCR screen of selected transcription-factor genes**. Provided information includes: AGI numbers, primer sequences, amplicon sizes, and C_T _values for 69 transcription-factor genes analyzed in the secondary qRT-PCR screen. Click here for file

Additional file 4 **Validation of the primary and secondary qRT-PCR screens with promoter-fusion analyses**. Venn diagram illustrating a significant overlap between the genes validated using the secondary qRT-PCR screen and the promoter-fusion analyses. Click here for file

Additional file 5 **Summary of promoter-fusion analyses**. Expression patterns of 28 *promoter:GFP *fusions representing 24 transcription-factor genes in the female gametophyte and during early seed development. Click here for file

Additional file 6 **Secondary expression patterns of transcription-factor *promoter:cGFP/n1GFP/n2GFP *fusions in the female gametophyte**. Epifluorescence images (A-I) were obtained from mature female gametophytes at 1DAE. Solid arrows indicate consistent expression patterns. Open arrows indicate expression patterns observed in rare instances (see Additional File 5 for details). (A) *pAT1G35520:n1GFP *expression in the antipodal cells and the synergid cells. (B) *pAT1G49770:cGFP *expression in the central cell, the synergid cells, and the egg cell. (C) *pAT1G55600:n1GFP *expression in the central cell. (D) *pAT3G01030:n2GFP *expression in the synergid cells, the central cell, and the egg cell. (E) *pAT5G01860:cGFP *expression in the central cell, the egg cell, the synergid cells, and the antipodal cells. (F) *pAT5G27880:n1GFP *expression in the central cell and the antipodal cells. (G) *pAT5G41090:n2GFP *expression in the cytoplasm of the antipodal cells. (H) *pAT5G45980:n1GFP *expression in the egg cell and the synergid cells. (I) *pAT5G56200:n1GFP *expression in the antipodal cells, the central cell, the egg cell, and the synergid cells. ac, antipodal cells; acn, antipodal cell nuclei; cc, central cell; ccn, central cell nucleus; ec, egg cell; ecn, egg cell nucleus; sc, synergid cells; scn, synergid cell nuclei. Scale bars: 50 μm. Click here for file

Additional file 7 **Secondary expression patterns of transcription-factor *promoter:n1GFP/n2GFP *fusions during early seed development**. Epifluorescence images (A-C) were obtained from developing seeds at flower stage 16. Arrows point to endosperm nuclei. Boxed areas indicate the micropylar location of the embryo. (A) *pAT3G01030:n2GFP *expression in stage III endosperm and in sporophytic cells of the integument and funiculus. (B) *pAT5G01380:n1GFP *expression in the zygote. (C) *pAT1G60280:n1GFP *expression in stage V endosperm and the pro-embryo. Scale bars: 50 μm. Click here for file

Additional file 8 **Comparison of data obtained in this study with data obtained from previously reported microarray-based screens**. Venn diagram illustrating the limited overlap between the transcription-factor genes identified in the five independent studies. Click here for file

Additional file 9 qRT-PCR analysis of *MYB98 *in seven pairs of *ms1*-*dif1 *ovary RNAs used in this study. Click here for file

Additional file 10 **Construction of promoter fusions for selected transcription-factor genes**. Provided information includes primer sequences used for constructing promoter fusions. Click here for file
