# Supplementary figures and images for: Identification of transcription-factor genes expressed in the Arabidopsis female gametophyte
Source: BMC Plant Biol. 2010 Jun 16;10:110. doi: 10.1186/1471-2229-10-110 (PMC3236301; doi:10.1186/1471-2229-10-110)

A

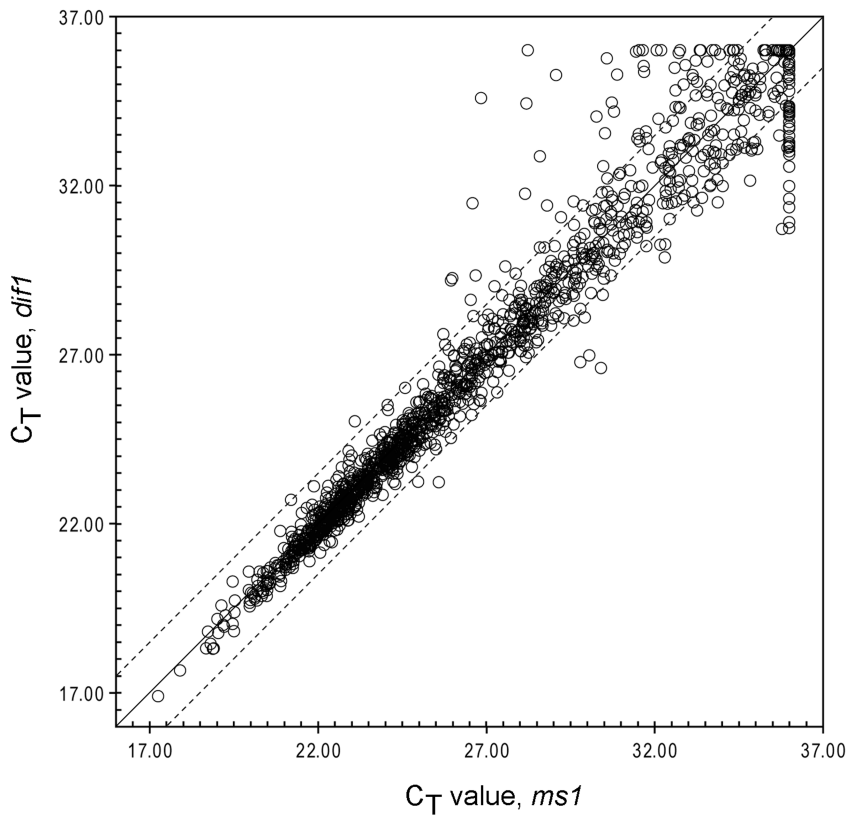

B

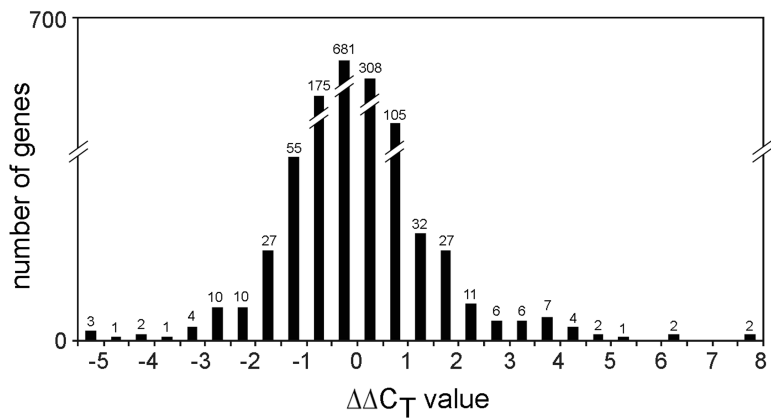

Supplement: Additional file 2 — Summary of the primary qRT-PCR screen. (A) Comparison of mRNA levels in ms1 and dif1 ovaries. Average CT values for the ms1 and dif1 ovary RNAs were compared for each transcription-factor gene analyzed. Dashed lines indicate the threshold of the primary screen (ΔΔCT > 1.5 or ΔΔCT < -1.5). (B) Histogram illustrating the distribution of ΔΔCT values obtained for all transcription-factor genes in the primary screen. [file 1471-2229-10-110-S2.PDF]

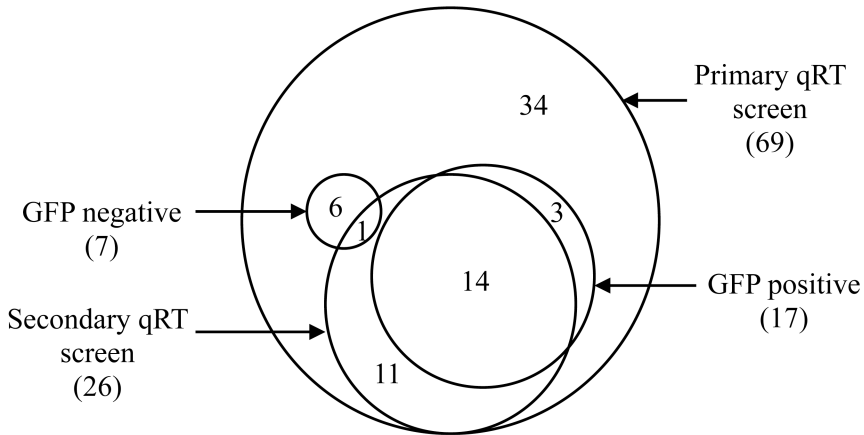

Supplement: Additional file 4 — Validation of the primary and secondary qRT-PCR screens with promoter-fusion analyses. Venn diagram illustrating a significant overlap between the genes validated using the secondary qRT-PCR screen and the promoter-fusion analyses. [file 1471-2229-10-110-S4.PDF]

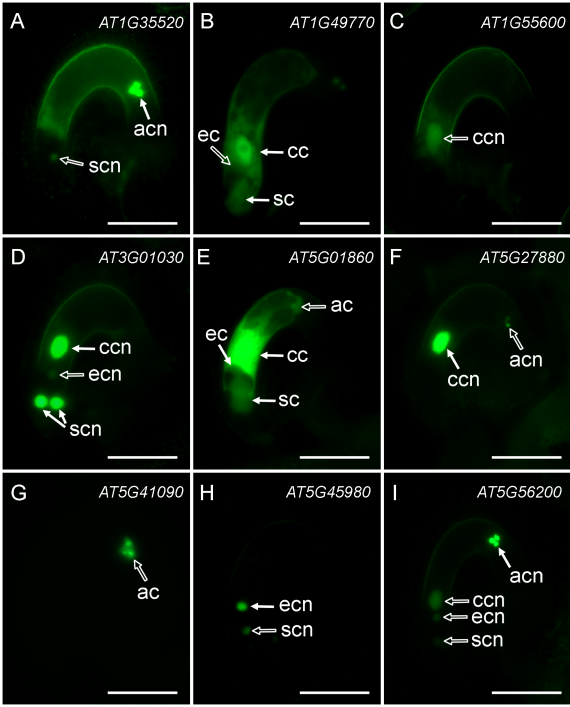

Supplement: Additional file 6 — Secondary expression patterns of transcription-factor promoter:cGFP/n1GFP/n2GFP fusions in the female gametophyte. Epifluorescence images (A-I) were obtained from mature female gametophytes at 1DAE. Solid arrows indicate consistent expression patterns. Open arrows indicate expression patterns observed in rare instances (see Additional File 5 for details). (A) pAT1G35520:n1GFP expression in the antipodal cells and the synergid cells. (B) pAT1G49770:cGFP expression in the central cell, the synergid cells, and the egg cell. (C) pAT1G55600:n1GFP expression in the central cell. (D) pAT3G01030:n2GFP expression in the synergid cells, the central cell, and the egg cell. (E) pAT5G01860:cGFP expression in the central cell, the egg cell, the synergid cells, and the antipodal cells. (F) pAT5G27880:n1GFP expression in the central cell and the antipodal cells. (G) pAT5G41090:n2GFP expression in the cytoplasm of the antipodal cells. (H) pAT5G45980:n1GFP expression in the egg cell and the synergid cells. (I) pAT5G56200:n1GFP expression in the antipodal cells, the central cell, the egg cell, and the synergid cells. ac, antipodal cells; acn, antipodal cell nuclei; cc, central cell; ccn, central cell nucleus; ec, egg cell; ecn, egg cell nucleus; sc, synergid cells; scn, synergid cell nuclei. Scale bars: 50 μm. [file 1471-2229-10-110-S6.PDF]

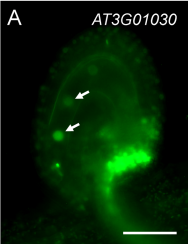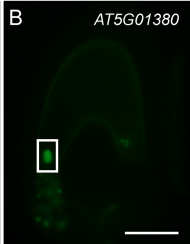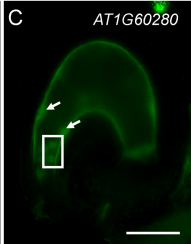

Supplement: Additional file 7 — Secondary expression patterns of transcription-factor promoter:n1GFP/n2GFP fusions during early seed development. Epifluorescence images (A-C) were obtained from developing seeds at flower stage 16. Arrows point to endosperm nuclei. Boxed areas indicate the micropylar location of the embryo. (A) pAT3G01030:n2GFP expression in stage III endosperm and in sporophytic cells of the integument and funiculus. (B) pAT5G01380:n1GFP expression in the zygote. (C) pAT1G60280:n1GFP expression in stage V endosperm and the pro-embryo. Scale bars: 50 μm. [file 1471-2229-10-110-S7.PDF]

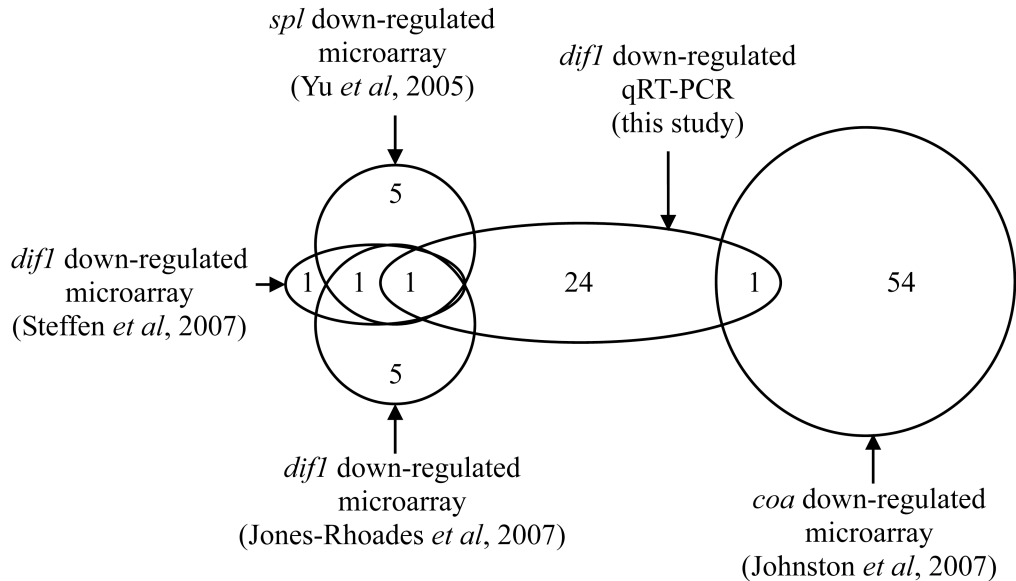

Supplement: Additional file 8 — Comparison of data obtained in this study with data obtained from previously reported microarray-based screens. Venn diagram illustrating the limited overlap between the transcription-factor genes identified in the five independent studies. [file 1471-2229-10-110-S8.PDF]
